# Consolidated Framework for Collaboration Research derived from a systematic review of theories, models, frameworks and principles for cross-sector collaboration

**DOI:** 10.1371/journal.pone.0244501

**Published:** 2021-01-04

**Authors:** Larissa Calancie, Leah Frerichs, Melinda M. Davis, Eliana Sullivan, Ann Marie White, Dorothy Cilenti, Giselle Corbie-Smith, Kristen Hassmiller Lich

**Affiliations:** 1 Friedman School of Nutrition Science and Policy, Tufts University, Boston, MA, United States of America; 2 Department of Health Policy and Management, Gillings School of Global Public Health, University of North Carolina at Chapel Hill, Chapel Hill, NC, United States of America; 3 Oregon Rural Practice-based Research Network, School of Medicine, Oregon Health and Science University, Portland, OR, United States of America; 4 Oregon Rural Practice-based Research Network, Oregon Health and Science University, Portland, OR, United States of America; 5 Department of Psychiatry, School of Medicine and Dentistry, University of Rochester Medical Center, Rochester, NY, United States of America; 6 Department of Maternal and Child Health, National Maternal and Child Health Workforce Development Center, Gillings School of Global Public Health, University of North Carolina at Chapel Hill, Chapel Hill, NC, United States of America; 7 Departments of Social Medicine and Internal Medicine, UNC Center for Health Equity Research, School of Medicine, University of North Carolina at Chapel Hill, Chapel Hill, NC, United States of America; Georgia Southern University, UNITED STATES

## Abstract

Cross-sector collaboration is needed to address root causes of persistent public health challenges. We conducted a systematic literature review to identify studies describing theories, models, frameworks and principles for cross-sector collaboration and synthesized collaboration constructs into the Consolidated Framework for Collaboration Research (CFCR). Ninety-five articles were included in the review. Constructs were abstracted from articles and grouped into seven domains within the framework: community context; group composition; structure and internal processes; group dynamics; social capital; activities that influence or take place within the collaboration; activities that influence or take place within the broader community; and activities that influence or take place both in the collaboration and in the community. Community engagement strategies employed by collaborations are discussed, as well as recommendations for using systems science methods for testing specific mechanisms of how constructs identified in the review influence one another. Researchers, funders, and collaboration members can use the consolidated framework to articulate components of collaboration and test mechanisms explaining how collaborations function. By working from a consolidated framework of collaboration terms and using systems science methods, researchers can advance evidence for the efficacy of cross-sector collaborations.

## Introduction

Collaboration across sectors has long been a strategy for addressing entrenched social problems such as addiction, environmental health justice, and health disparities [1–3]. Cross-sector collaborations are groups whose members represent different sectors in a community, such as healthcare, education, community residents, and government, who contribute their unique perspectives, resources, capabilities and social capital toward a shared vision that could not be achieved by organizations acting within a single sector [4, 5]. Recognizing that social determinants of health and other factors are influenced by many sectors, in 2019 the Robert Wood Johnson Foundation called for on-going collaborations between sectors to create healthy communities where all individuals can lead healthy lives [6]. The National Academy of Medicine, the Centers for Disease Control and Prevention, Centers for Medicaid and Medicare Services and health care systems such as Kaiser Permanente have all called for, and funded, cross-sector collaboration efforts to promote health and reduce disease in communities [7–10]. In addition, states like Oregon have implemented policies to support cross-sector collaborations between medical (hospital, primary care), public health, patients as a stakeholder group, and other community-based services providers (behavioral health, criminal justice, education) [11]. Cross-sector collaboration approaches are likely to continue being applied to complex social problems within communities.

A variety of theories, models, frameworks and principles for cross-sector collaborations are proposed in the scientific literature as well as through practitioner-oriented organizations and publications [12]. In 2002 Butterfoss and Kegler noted that “the practice of coalition building has outpaced the development of coalition theory” (p 161, [1]) and went on to propose an initial version of the Community Coalition Action Theory (CCAT) that integrated published and grey literature to describe the formation, maintenance, and function of coalitions in communities. Since then practitioners and researchers have expanded the repertoire of cross-sector collaboration frameworks used to plan, support, and evaluate such entities. Collective Impact, first proposed by Kania & Kramer in 2011 [13], has become particularly popular, despite some concerns that it does not acknowledge decades of cross-sector collaboration scientific literature and “misses the social justice core that exists in many coalitions” (p4, [14]). Some studies of Collective Impact report positive results [15, 16], while others report mixed findings and limitations of the model [17–19]. Practitioners, researchers, and funders would benefit from an analysis of commonalities between frameworks and an exploration of the community engagement strategies they employ to create change in their communities.

In order to advance the science of the processes through which cross-sector collaborations engage community members and influence change, the field needs a comprehensive view of existing frameworks as a step toward developing cross-sector collaboration theories that can guide research and practice. While several reviews of cross-sector collaboration studies have been conducted [2, 20, 21], they were conducted thirteen to twenty years ago. Cross-sector collaboration literature has expanded significantly since those reviews were conducted and thus an updated review is warranted. The purpose of our review is to inform cross-sector collaboration research and practice by identifying concepts and community engagement strategies in the literature that are relevant to cross-sector collaboration planning, implementation, and evaluation. Our objective is to provide a consolidated presentation of constructs with consistent terminology and definitions from across multiple theories and frameworks. Researchers and practitioners can select constructs and engagement strategies from our consolidated framework that are most relevant to their context and use them for further theory development and verification, evaluation of collaboration progress over time, and to help diagnose or explain variation in collaboration process and outcomes. In summary, we aimed to identify and describe constructs within theories, models, frameworks and principles for cross-sector collaborations published in the peer-reviewed scientific literature; document the community engagement approaches they employ; and synthesize constructs into a comprehensive framework. This thorough, up-to-date review provides a foundation for collaborations, funders, and researchers to practice, build upon, and rigorously test models of cross-sector collaboration.

## Methods

We conducted a systematic review using PRISMA guidelines to identify peer-reviewed publications describing theories, models, frameworks and principles (hereafter referred to as “models”) for cross-sector collaboration [22]. To synthesize these results, we created a conceptual framework–the Consolidated Framework for Collaboration Research (CFCR)—integrating the constructs for models identified in the review. “We” are a team of researchers who study approaches to addressing a variety of public health challenges, such as mental health concerns, chronic disease prevention and management, obesity prevention, cancer prevention, and maternal and child health concerns. We work with community members and groups and saw a need for a comprehensive model of how community collaborations operate in order to further study and inform community-based work.

### Search strategy

With assistance of a health science research librarian, we searched PubMed, Embase, and EBSCO (CINHAL Plus with Full Text and Social Work Abstracts) from date of database initiation to November 2016 for published cross-sector collaboration models. The first author met with the librarian to establish a specific search strategy that was likely to return articles that were relevant to the review. After discussing the goals of the review, we provided several articles that were illustrative of the types of articles we expected our review to return and worked with the librarian to develop a strategy to systematically identify relevant articles. Within that strategy, the librarian suggested databases to search, recommended searching variations on search terms, and advised on the search logic within each database in order to keep the search consistent across databases. We conducted a complicated search using 48 search terms, including ‘cross sector collaboration,’ ‘cross-sector collaboration,’ ‘cross-sector network,’ ‘multisector network,’ multi-system collaboration,’ ‘council,’ ‘coalition,’ ‘collective impact,’ ‘framework,’ ‘theory,’ and ‘model.’ A full list of search terms is available in S1 Table. Search results were merged and de-duplicated. Articles were excluded if they were not written in English; if the full text was not available; if they mentioned a collaboration but did not describe a generalizable model; referred to an existing model without adding or revising constructs; or described a collaboration within a single sector. Two authors reviewed all titles and abstracts for inclusion/exclusion and reconciled any disagreements. The full text of selected articles was then read by two authors to determine whether screened articles met the inclusion criteria. The search was updated in 2020 by repeating the search to include articles published between December 2016 and July 2020. One author reviewed all titles, abstracts, and full text to update the list of included articles.

### Data abstraction

Three authors created a data abstraction form and then revised the form based on input from the larger author group. We pilot-tested the revised abstraction form with the large group and further revised the form to create a final abstraction form. The final form was programmed into Qualtrics, an online survey platform, and contained a mix of multiple-choice format questions (e.g., What type of cross-sector collaboration does this article describe?) and open text boxes (e.g., What is the stated objective of cross-sector collaboration described in this article?) to abstract relevant information in each article. Two-member co-author teams abstracted text from included articles using the final form. One author abstracted the information from each included article and then another member reviewed the abstractions–adding to or editing the abstraction as needed. We used the five following major domains to guide text abstraction: constructs described in the model; definitions of “system”; organizational structure; community engagement activities; and evaluation descriptions. In addition, we abstracted details on the study design, collaboration type (e.g., coalition, council, collaborative as defined by the authors), topic(s) the collaboration focused on, objective(s) of the collaboration, geographic catchment area, sectors represented, collaboration stage, and any steps and specific actions that were recommended to support collaboration activities.

### Coding process

We analyzed abstracted text using content analysis [23]. Abstracted textual data were uploaded into Dedoose [24] and coded. The first author reviewed included articles and generated an initial codebook based on Allen and colleagues’ model [5] and Butterfoss and Kegler’s CCAT [25]. Allen’s model shows how internal capacity constructs, such as leadership and member empowerment relate to collaboratives’ goal of changing systems through institutionalized policies and practices [5]. The CCAT is a theory that contains similar constructs to Allen’s model, but includes stages of coalition formation, implementation of strategies, and community health outcomes [25]. CCAT and Allen’s model were selected because they can be applied to a range of public health challenges and have been empirically tested with coalitions [5, 26, 27].

Two authors pilot-tested the codebook by coding abstracted text from 10 randomly selected articles using the initial codebook. Testing and refining a codebook is recommended when conducting qualitative analysis with a team of researchers [28]. They met to discuss how they applied codes and opportunities to revise the codebook in order to capture relevant concepts across a range of article types. Based on the pilot-test, we refined code definitions, added new codes, and removed or consolidated redundant codes. Subsequently, the two authors coded additional sets of 10 articles using the revised codebook until they reached at least 65% agreement for each category within the codebook. Percent agreement ranged from 67–100% with an average of 84% agreement. Then the first author coded all definitions of “system”; organizational structure; community engagement activities; and descriptions of evaluation. Two authors double-coded constructs, then the research team members reconciled discrepancies by discussing the rationale behind applied codes and selecting an agreed upon code(s) for each excerpt. Final codes and definitions are in Table 2.

### Analysis and synthesis

We calculated code frequencies for abstracted text that could be categorized and counted (e.g., collaboration type, focus area, sectors represented) and synthesized our findings. Using an iterative process, we grouped and synthesized the coded constructs into a conceptual model called the Consolidated Framework for Collaboration Research (CFCR), to visually show the frequency with which constructs were abstracted from included articles and to hypothesize how groups of constructs might relate to one other. The CFCR is inspired by the Consolidated Framework for Implementation Research that was similarly developed through a literature review and sought to inventory and consolidate constructs within the implementation field [29]. CCAT, Allen’s model, and findings from this review informed CFCR. Constructs that occurred in five percent or more of the articles included in this review are included in the framework.

## Results

### Included articles

**Fig 1:**
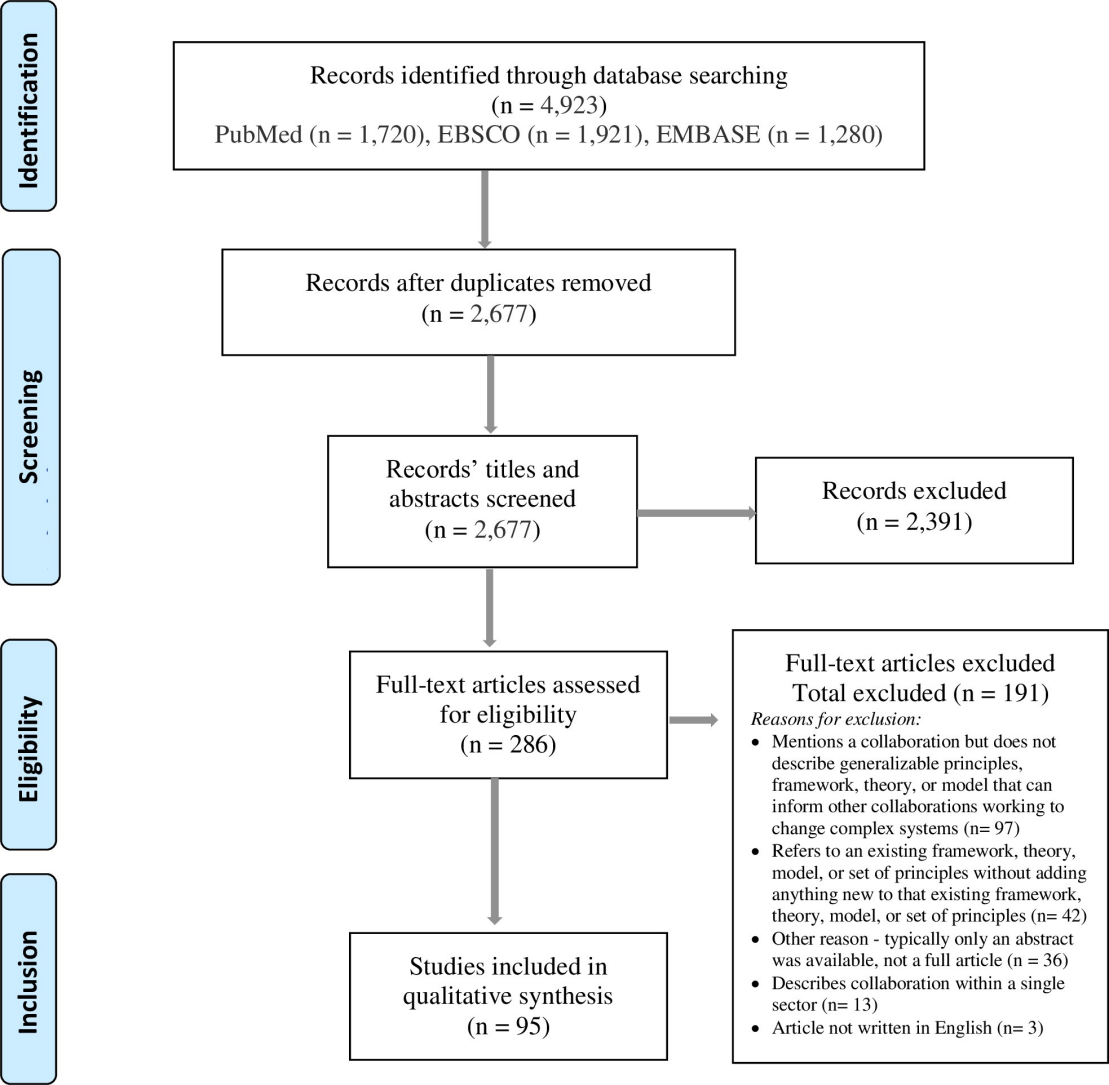
PRISMA diagram showing review search results, included articles, excluded articles and reasons for article exclusion.

A total of 4,923 articles were identified across the three databases searched, resulting in 2,677 unique articles (Fig 1). We reviewed the full text of 286 articles; 95 (33%) articles met inclusion criteria. Most articles excluded during the full text review mentioned a collaboration but did not describe generalizable models that can inform other collaborations (51%) or referred to existing theories, models, frameworks and principles and did not make significant modifications to the model (22%).

### Study characteristics

As detailed in Table 1, included articles used diverse research designs and addressed a variety of topics. Over half of the articles were case studies or lessons from the field (57%). Cross-sectional studies of one or more collaborations were the next most common study type (26%) followed by conceptual papers, which reviewed the literature and proposed a new model (12%); two articles (2%) described trials where community-level outcomes were evaluated. Topics addressed included healthcare access, broad community health, and other specific disease or health-related foci (e.g., obesity, teen pregnancy). Promoting health, improving health systems, and reducing substance abuse were the most common topics.

**Table 1:** Study types and descriptions of collaborations presented in reviewed articles.

Author, Year	Title	Collaboration Type	Topic	Geographic unit of influence	Study Design
Allen, 2012 [5]	"Changing the Text": Modeling Council Capacity to Produce Institutionalized Change	Council	Intimate partner violence	Region	Cross-sectional analysis
Amed, 2015 [38]	Creating a collective impact on childhood obesity: Lessons from the SCOPE initiative	Collaborative	Childhood obesity	Community	Case study, report from the field or lessons learned
Baranowski, 1982 [62]	Agency coalitions for targeted service delivery: foiled designs, failed development, but final delight	Coalition	Low birth weight	State or province	Case study, report from the field or lessons learned
Barnes, 2017 [47]	Functional Characteristics of Health Coalitions in Local Public Health Systems: Exploring the Function of County Health Councils in Tennessee	Coalition	Promote health	County	Cross-sectional analysis
Behringer, 2010 [63]	Models for local implementation of comprehensive cancer control: meeting local cancer control needs through community collaboration	Coalition	Cancer	Other	Case study, report from the field or lessons learned
Bertam, 2008 [46]	Establishing a Basis for Multi-System Collaboration: Systemic Team Development	Collaborative	Improve health and human services systems	City or municipality	Case study, report from the field or lessons learned
Brady, 2014 [64]	Integrating the Life Course into MCH Service Delivery: From Theory to Practice	Coalition	Infant mortality & child development	City or municipality	Case study, report from the field or lessons learned
Braithwaite, 1989 [65]	Community Organization and Development for Health Promotion Within an Urban Black Community: A Conceptual Model	Other	Community empowerment	Community	Case study, report from the field or lessons learned
Butterfoss, 1998 [66]	CINCH: An Urban Coalition for Empowerment and Action	Coalition	Childhood immunizations	City or municipality	Case study, report from the field or lessons learned
Butterworth, 2017 [67]	Partnerships in Employment: Building strong coalitions to facilitate systems change for youth and young adults	Coalition	Increasing employment for youth with intellectual and developmental disabilities	State or province	Case study, report from the field or lessons learned
Carman, 2018 [68]	Cross Jurisdictional Boundaries to Build a Health Coalition: A Kentucky Case Study	Coalition	Health promotion	Region	Case study, report from the field or lessons learned
Choy, 2016 [69]	Examining the role of a community coalition in facilitating policy and environmental changes to promote physical activity: the case of Get Fit Kaua'i	Coalition	Physical activity	County	Case study, report from the field or lessons learned
Chutuape, 2015 [48]	A Tailored Approach to Launch Community Coalitions Focused on Achieving Structural Changes: Lessons Learned from a HIV Prevention Mobilization Study	Coalition	HIV	City or municipality	Cross-sectional analysis
Clark, 2006 [70]	Community Coalitions to Control Chronic Disease: Allies Against Asthma as a Model and Case Study	Coalition	Asthma	City or municipality	Conceptual paper
Courie, 2014 [49]	Managing Public Health in the Army Through a Standard Community Health Promotion Council Model	Council	Strengthen public health systems	Other	Case study, report from the field or lessons learned
Cramer, 2006 [71]	A Conceptual Model for Understanding Effective Coalitions Involved in Health Promotion Programing	Coalition	Chronic disease	Other	Conceptual paper
Cooper, 2019 [37]	Justice System Reform for Health Equity: A Mixed Methods Examination of Collaborating for Equity and Justice Principles in a Grassroots Organizing Coalition	Coalition	Justice reform	City or municipality	Case study, report from the field or lessons learned
Davidson, 2010 [72]	Creating a Provincial Family Council to Engage Youth and Families in Child & Youth Mental Health Systems	Council	Mental health care for children/youth	State or province	Case study, report from the field or lessons learned
de Montigny, 2019 [12]	The fundamentals of cross-sector collaboration for social change to promote population health	Other	Promote health	Other	Conceptual paper
Diehl, 2005 [73]	The school community council: creating an environment for student success	Council	Education	Neighborhood	Case study, report from the field or lessons learned
Downey, 2008 [30]	Defining elements of success: a critical pathway of coalition development	Coalition	Injury prevention	County	Conceptual paper
Dunlop, 2001 [74]	Inside-outside: boundary-spanning challenges in building rural health coalitions	Coalition	Teenage pregnancy	Region	Case study, report from the field or lessons learned
Edwards, 2013 [75]	Development and evaluation of a "working together" framework and a tool kit to enhance inter-organizational relationships in healthcare	Other	Improve health system	Other	Cross-sectional analysis
Ehrlich, 2015 [76]	Integrating collaborative place-based health promotion coalitions into existing health system structures: the experience from one Australian health coalition	Coalition	Chronic disease	Community	Case study, report from the field or lessons learned
Fagan, 2016 [58]	Patients, Persistence, and Partnership: Creating and Sustaining Patient and Family Advisory Councils in a Hospital Setting	Council	Improve care delivery	Other	Case study, report from the field or lessons learned
Feinberg, 2004 [77]	Readiness, Functioning, and Perceived Effectiveness in Community Prevention Coalitions: A Study of Communities That Care	Coalition	Promote health in adolescents	County	Cross-sectional analysis
Felland, 2011 [50]	Improving health care access for low-income people: Lessons from ascension health's community collaboratives	Collaborative	Improve health system	City or municipality	Case study, report from the field or lessons learned
Fisher, 1996 [39]	Acceptability and Feasibility of a Community Approach to Asthma Management: The Neighborhood Asthma Coalition (NAC)	Coalition	Asthma	Neighborhood	Case study, report from the field or lessons learned
Fleury, 2014 [78]	The role of advocacy coalitions in a project implementation process: The example of the planning phase of the At Home/Chez Soi project dealing with homelessness in Montreal	Coalition	Homelessness & mental health	City or municipality	Case study, report from the field or lessons learned
Flewelling, 2016 [79]	Assessing Community Coalition Capacity and its Association with Underage Drinking Prevention Effectiveness in the Context of the SPF SIG	Coalition	Substance abuse & promoting health in adolescents	State or province	Trial
Flood, 2015 [80]	The Collective Impact Model and Its Potential for Health Promotion: Overview and Case Study of a Healthy Retail Initiative in San Francisco	Coalition	Healthy food access, & reduce tobacco and alcohol availability	Neighborhood	Case study, report from the field or lessons learned
Foster-Fishman, 2001 [21]	Building Collaborative Capacity in Community Coalitions: A Review and Integrative Framework	Coalition	Not specified	Not described	Conceptual paper
Galvez, 2019 [81]	Building New York State Centers of Excellence in Children’s Environmental Health: A Replicable Model in a Time of Uncertainty	Network	Health promotion in children	State or province	Case study, report from the field or lessons learned
Giachello, 2003 [34]	Reducing diabetes health disparities through community-based participatory action research: The Chicago Southeast diabetes community action coalition	Coalition	Health disparities	Community	Case study, report from the field or lessons learned
Gomez, 2005 [82]	Sustainability of community coalitions: an evaluation of communities that care	Coalition (multiple)	Promote health in adolescents	Community	Conceptual paper
Green, 2014 [83]	Cross-sector collaborations in Aboriginal and Torres Strait Islander childhood disability: a systematic integrative review and theory-based synthesis	Collaborative	Reduce child disability disparities	Other	Literature review
Hanson, 2016 [84]	Testing the Community-Based Learning Collaborative (CBLC) implementation model	Collaborative	Mental health care for children	Community	Cross-sectional analysis
Hardy, 2013 [85]	A model for evaluating the activities of a coalition-based policy action group: the case of Hermosa Vida	Coalition	Childhood obesity	Community	Case study, report from the field or lessons learned
Horne, 2013 [40]	Implementing the ACHIEVE Model to Prevent and Reduce Chronic Disease in Rural Klickitat County, Washington	Coalition	Obesity & chronic disease	County	Case study, report from the field or lessons learned
Huberty, 2010 [86]	From good ideas to actions: A model-driven community collaborative to prevent childhood obesity	Collaborative	Childhood obesity	City or municipality	Case study, report from the field or lessons learned
Hupert, 2015 [51]	Optimizing Health Care Coalitions: Conceptual Frameworks and a Research Agenda	Coalition	Improve health systems & disaster preparedness	Other	Conceptual paper
Jenkins, 2011 [35]	Efforts to Decrease Diabetes-Related Amputations in African Americans by the Racial and Ethnic Approaches to Community Health Charleston and Georgetown Diabetes Coalition	Coalition	Reduce health disparities among patients with diabetes	Other	Case study, report from the field or lessons learned
Johnson, 2009 [41]	Building Community Participatory Research Coalitions from the Ground Up: The Philadelphia Area Research Community Coalition	Coalition	Health disparities	City or municipality	Case study, report from the field or lessons learned
Kegler, 2005 [87]	Mobilizing communities for teen pregnancy prevention: Associations between coalition characteristics and perceived accomplishments	Coalition	Teenage pregnancy	Community	Cross-sectional analysis
Kegler, 2012 [88]	Advancing coalition theory: the effect of coalition factors on community capacity mediated by member engagement	Coalition	Promote health	Community	Case study, report from the field or lessons learned
Ken-Opurum, 2020 [89]	Assessing Rural Health Coalitions Using the Public Health Logic Model: A Systematic Review	Coalition	Health disparities	Other	Conceptual paper
Koelen, 2012 [90]	The healthy alliances (HALL) framework: prerequisites for success	Alliance	Chronic disease	Not described	Conceptual paper
Korn, 2018 [91]	Engaging Coalitions in Community-Based Childhood Obesity Prevention Interventions: A Mixed Methods Assessment	Coalition	Childhood obesity	Other	Literature review
Kramer, 2005 [59]	Coalition models: Lessons learned from the CDC's Community Coalition Partnership Programs for the Prevention of Teen Pregnancy	Coalition	Family planning	City or municipality	Cross-sectional analysis
Kreger, 2011 [92]	Creating an Environmental Justice Framework for Policy Change in Childhood Asthma: A Grassroots to Treetops Approach	Coalition	Asthma	Community	Case study, report from the field or lessons learned
Kristjansson, 2020 [45]	Implementing the Icelandic Model for Preventing Adolescent Substance Use	Coalition	Substance abuse	Neighborhood	Case study, report from the field or lessons learned
Kubik, 2001 [42]	A practical, theory-based approach to establishing school nutrition advisory councils	Council	Promote health in schools	Other	Trial
Kumpfer, 1993 [33]	Leadership and team effectiveness in community coalitions for the prevention of alcohol and other drug abuse	Coalition	Substance abuse	County	Cross-sectional analysis
Lara, 2006 [52]	Improving quality of care and promoting health care system change: The role of community-based coalitions	Coalition	Integrated service delivery for asthma	Community	Case study, report from the field or lessons learned
Laraia, 2003 [93]	A Framework for Assessing the Effectiveness of Antihunger Advocacy Organizations	Other	Hunger	State or province	Case study, report from the field or lessons learned
Lewis, 2011 [94]	Transforming the urban food desert from the grassroots up: A model for community change	Other	Health disparities	Other	Case study, report from the field or lessons learned
Loh, 2016 [95]	Coalition de Salud Comunitaria (COSACO): using a Healthy Community Partnership framework to integrate short-term global health experiences into broader community development	Coalition	Promote health	City or municipality	Case study, report from the field or lessons learned
Marchand, 2006 [96]	Building successful coalitions to promote advance care planning	Coalition	Advance care planning	State or province	Cross-sectional analysis
McClure, 1983 [97]	School advisory council participation and effectiveness	Council	Improving education	Community	Cross-sectional analysis
McFall, 2004 [98]	A qualitative evaluation of rural community coalitions	Coalition	Community development & improve health systems	County	Case study, report from the field or lessons learned
Metzger, 2005 [99]	The Effects of Leadership and Governance Processes on Member Participation in Community Health Coalitions	Coalition	Improve care delivery	Other	Cross-sectional analysis
Mulroy, 1997 [100]	Building a neighborhood network: interorganizational collaboration to prevent child abuse and neglect	Other	Prevent child abuse and neglect	Community	Cross-sectional analysis
Nicola, 2005 [53]	Turning Point's National Excellence Collaboratives: Assessing a New Model for Policy and System Capacity Development	Collaborative	Strengthen public health systems	Other	Case study, report from the field or lessons learned
Norris, 2000 [101]	The Healthy Communities Movement and the Coalition for Healthier Cities and Communities	Coalition	Promote health	Community	Case study, report from the field or lessons learned
Nowell, 2011 [31]	Examining Multi-Sector Community Collaboratives as Vehicles for Building Organizational Capacity	Collaborative	Reduce domestic violence	County	Cross-sectional analysis
O'Neill, 1997 [102]	Coalition theory as a framework for understanding and implementing intersectoral health-related interventions	Coalition (sort of); Other	Promote health	City or municipality	Case study, report from the field or lessons learned
Palafox, 2018 [103]	A Socio-Ecological Framework for Cancer Control in the Pacific: A Community Case Study of the US Affiliated Pacific Island Jurisdictions 1997–2017	Network	Cancer control	Region	Case study, report from the field or lessons learned
Packard, 2013 [54]	Implementing Services Integration and Interagency Collaboration: Experiences in Seven Counties	Other	Improve health system	County	Cross-sectional analysis
Paine-Andrews, 1997 [104]	Community coalitions to prevent adolescent substance abuse: The case of the 'project freedom' replication initiative	Coalition	Substance abuse gang violence	County	Case study, report from the field or lessons learned
Pierre, 2020 [105]	Building a Culture of Health at the Neighborhood Level Through Governance Councils	Council	Promote health	Neighborhood	Cross-sectional analysis
Polivka, 1995 [106]	A conceptual model for community interagency collaboration	Other	Promote health	Community	Conceptual paper
Powell, 2014 [60]	Pathways to effectiveness in substance abuse prevention: Empowering organizational characteristics of community-based coalitions	Coalition	Substance abuse	Community	Cross-sectional analysis
Powell, 2017 [61]	Empowerment in Coalitions Targeting Underage Drinking: Differential Effects of Organizational Characteristics for Volunteers and Staff	Coalitions	Substance abuse	Region	Cross-sectional analysis
Revell, 2011 [107]	Applying the performance partnership model to smoking cessation: lessons learned by the smoking cessation leadership center	Coalition	Tobacco use	Other	Case study, report from the field or lessons learned
Rosenthal, 2006 [55]	The Coalition Process at Work: Building Care Coordination Models to Control Chronic Disease	Coalition	Integrated service delivery for asthma	Other	Case study, report from the field or lessons learned
Salem, 2005 [56]	MAPP in Chicago: A Model for Public Health Systems Development and Community Building	Coalition	Strengthen public health systems	Community	Case study, report from the field or lessons learned
Sánchez, 2015 [108]	New Mexico Community Health Councils: Documenting Contributions to Systems Changes	Council	Promote health	Other	Case study, report from the field or lessons learned
Shapiro, 2013 [43]	Measuring Dimensions of Coalition Functioning for Effective and Participatory Community Practice	Coalition	Youth development & promote health in adolescents	Community	Cross-sectional analysis
Sharma, 2016 [109]	"How Can We Talk about Patient-centered Care without Patients at the Table" Lessons Learned from Patient Advisory Councils	Council	Increase patient engagement	Other	Cross-sectional analysis
Shenson, 2008 [110]	Expanding the Delivery of Clinical Preventive Services Through Community Collaboration: The SPARC Model	Coalition	Vaccination and cancer screening	County	Case study, report from the field or lessons learned
Silverman, 2015 [111]	"Collaborating for consensus: Considerations for convening Coalition stakeholders to promote a gender-based approach to addressing the health needs of sex workers"	Coalition	Gender disparities	State or province	Case study, report from the field or lessons learned
Smith, 2007 [32]	Multi-level influences on the practice of inter-agency collaboration in child welfare and substance abuse treatment	Other	Improve health and human services systems & substance abuse	State or province	Cross-sectional analysis
Stevens, 2007 [112]	Children's health initiatives in California: The experiences of local coalitions pursuing universal coverage for children	Coalition	Health insurance coverage for children	County	Cross-sectional analysis
Teaster, 2010 [113]	Kentucky's local elder abuse coordinating councils: A model for other states	Council	Prevent elder abuse	Community	Cross-sectional analysis
Thompson, 2002 [44]	A Collaboration Model for Enhanced Community Participation	Collaborative	Community development	Neighborhood	Case study, report from the field or lessons learned
Towe, 2016 [114]	Cross-sector collaborations and partnerships: Essential ingredients to help shape health and well- being	Other	Obesity & chronic disease	Other	Case study, report from the field or lessons learned
Travis, 2011 [115]	The Community Action Framework in Practice: An Illustration Based on the Ready by 21 Coalition of Austin/Travis County	Coalition	Youth development	County	Case study, report from the field or lessons learned
Tseng, 2011 [116]	Moving toward being analytical: a framework to evaluate the impact of influential factors on interagency collaboration	Other	Not specified	Not described	Conceptual paper
Tucker, 2006 [36]	The REACH 2010 logic model: an illustration of expected performance	Coalition	Health disparities	Community	Conceptual paper
Valentijn, 2015 [117]	Exploring the success of an integrated primary care partnership: a longitudinal study of collaboration processes	Other	Integrated service delivery	Other	Cross-sectional analysis
Walter, 2000 [118]	A Template for Family-Centered Interagency Collaboration	Other	Improve health and human services systems	Community	Case study, report from the field or lessons learned
Wandersman, 1996 [119]	Toward a social ecology of community coalitions	Coalition	Substance abuse	Community	Case study, report from the field or lessons learned
Watson-Thompson, 2008 [120]	A Framework for Community Mobilization to Promote Healthy Youth Development	Council	Youth development	Neighborhood	Case study, report from the field or lessons learned
Weiner, 2002 [57]	Management and Governance Processes in Community Health Coalitions: A Procedural Justice Perspective	Coalition	Improve health system	Community	Cross-sectional analysis
Wunrow, 2001 [121]	Promoting Youth/Adult Partnerships: The Seven Circles Coalition in Sitka, Alaska	Coalition	Substance abuse & youth development	Region	Case study, report from the field or lessons learned

The geographic scope that collaborations were working to influence was described in 72 articles (76%). “Community” was the most frequently mentioned geographic target area (24%), followed by counties (15%), cities or municipalities (14%), state or province-level focus areas (11%), neighborhood (7%), and regional (6%). The number of sectors involved in collaborations ranged from two to ten, including social services, public health, education, criminal justice, public safety, government, healthcare, military, housing, faith organizations, and community members. Healthcare (57%), government (37%), and community-based organizations (35%) were the most common sectors included in collaborations. Caregivers (4%), military (2%), and transportation (2%) were the least frequently mentioned sectors. Described cross-sector collaborations spanned the formation, maintenance, and institutionalization stages of collaboration, with many articles applicable to multiple stages. Articles described a variety of collaborative objectives including coordinate a system or multi-sector response to complex issues [30–33] such as health disparities [34–37]; engage community in multi-sector approaches to change [38–45]; avoid duplicating efforts to address a complex problem [46, 47]; work together to create structural change [48]; build public health or health care infrastructure and coordination [49–57]; institutionalize partnerships [58]; mobilize resources [59]; and implement multi-sector programs and policies [60, 61].

### Construct code results

Construct code results are presented in Table 2, including construct code names, percent of articles containing each construct, and construct definitions. Sample article excerpts for each construct are presented in S2 Table. Articles often described collaboration goals in terms of improving a system and/or community-level outcome(s) related to health. The most commonly applied construct codes were “broad, active membership” (construct code contained in 61% of articles), followed by “interventions” (58%), “organizational structure and processes” (51%), and “shared vision” (51%). These are arguably defining features of collaborations, which were repeatedly described as being composed of members that work together through formal and informal processes to apply their perspective and experience to build a future that the groups agree is better in some specific ways than the current state. About 30% of articles acknowledged that the context in which a cross-sector collaboration is working matters. Some articles (12–14%) recommended or reported that collaborations sought to learn about specific contexts, such as political or economic contexts. Cross-sector collaborations undertake activities that operate within the collaboration, such as planning, and externally to the collaboration, often in partnership with communities. Examples of external activities are needs assessments and community education. Activities keep collaboration members engaged, build credibility within their communities, and move the collaboration toward realizing its goals. More than half of the articles described community engagement approaches, indicating that community engagement is a common element of cross-sector collaborations. Community representation within collaboratives was critical in many of the identified studies. Additional strategies to engage community members included seeking input about collaboration priorities directly from community members, community mobilizing around specific initiatives, offering training and capacity building opportunities for community members, and involving community members in data collection or implementation activities. Primary data collection from community members, including focus groups, surveys, and interviews, was mentioned in 20% of articles.

**Table 2:** Construct codes, percent of articles containing each construct code, and sample construct excerpts or excerpt summaries from articles included in the review.

Construct code	% of articles containing code	Brief Definition
**Community context**		
Community context	27%	General analysis of, acknowledgement of, or appreciation for the community context in which the collaboration is operating
Political context	14%	Analysis of, or appreciation for the political context in which the collaboration is operating
Economic context	13%	Analysis of, or appreciation for the economic context in which the collaboration is operating
Social context	12%	Analysis of, or appreciation for the social context in which the collaboration is operating
Cultural context	12%	Analysis of, or appreciation for the cultural context in which the collaboration is operating
**Group composition**		
Breadth of active membership	61%	Mention of broad membership, or “strategies for ensuring diverse member representation, including recruitment, by-laws requiring broad membership, flexible meeting times, examining “who is missing” and reaching out”
Broad representation	31%	Reference to professionals or members of organizations that work to address the focal problem of the collaboration; Broad representation (as members or advisors) from many sectors involved in the problem the collaborative is trying to address
Community representation	23%	Individuals who experience the issues being addressed are represented on the collaboration
**Structure and internal processes**		
Organizational structure and processes	51%	Description of formal and non-formal processes that govern collaboration interactions, roles, and activities
Shared vision	51%	Attention to the need for a common vision, mission, or guiding principles to be shared by collaboration members
Funding	33%	Description of need for, or use of financial resources to support the collaboration’s work
Internal communication	32%	Communication that takes place within the collaborative
Leadership	29%	Mentions leadership, but doesn’t describe a particular leadership style
Executive committee	27%	Mention of an executive committee, which could include president, VP, secretary
Stages of collaboration	21%	Description of specific stages that collaborations progress through, though not necessarily linearly
Distributive/ empowerment leadership	21%	A leadership style that empowers others to speak up, learn, take on leadership roles themselves
Working group(s)	20%	A subset of members that focus on a particular issues, such as a clinical improvement working group or policy committee
Staff/admin support	14%	Mention of staff or administrative support (paid or unpaid official coordinator who performs administrative and/or communication tasks)
Flexibility	11%	Ability to adapt to or accommodate changing circumstances
Parallel working groups	7%	The coalition addresses some tasks through parallel work groups with the goal of efficiently using collaboration resources and expertise
Adherence to a well-defined plan or best practice	6%	Extent to which what the collaboration does adheres to a best practice or well-defined implementation plan
**Social capital**		
Knowledge sharing	40%	Members gain knowledge of the systems and issues they are trying to address through their membership in the collaboration
Relationships	31%	Members form bonds between individuals and organizations through their interactions in the collaboration
Member empowerment	26%	Members feel empowered to address complex problems in their community, inside and outside the collaborative
Access to resources	21%	Collaboration or its members have more access to grants, funding, students, volunteers, experts, etc. than they would have if the collaboration didn’t exist
Credibility	11%	The collaboration is viewed as a trust-worthy organization within the community
**Group dynamics**		
Collaboration climate	32%	Extent to which collaboration members can work together without significant interpersonal conflict
Trust	18%	Members develop trust with other members
Members’ influence on decision-making	16%	Members’ perceptions about their ability to provide input and have power within group decision
Commitment-rewards balance	16%	Balancing collaboration commitments with rewards to create a win-win for participating
Balanced participation	16%	Members feel that work is shared fairly; they are not overly burdened to “pull the weight” of the collaboration
Members’ satisfaction with collaboration	13%	Members express feeling their participation is valuable/useful and/or that the collaboration is meeting their expectations
Perceived fairness	6%	Ability to resolve conflicts and conduct business such that involved parties feel fairly treated
Accountability	5%	Members are motivated to participate or carry through activities in order to satisfy the larger group
**Activities that influence or take place in the community**		
Interventions	58%	Programs, policies, initiatives, campaigns, activities that the collaboration conducts to address a focal problem
Needs assessments	42%	Reference to an attempt by the collaboration to understand the needs of the communities or context in which they are working to improve outcomes
Planning	33%	Description of planning activities, or intentions to plan. Excludes strategic thinking, which is more about where the collaboration is, where it is going, and how to get there
Data collection	20%	Description of focus groups, interviews, surveys or other primary data collection from community members and organizations
Mutually reinforcing activities	14%	A set of interventions are selected and supported by subsets of the collaboration together with external partners based on overall alignment/synergy
Support other organizations’ initiatives	5%	Mentions lending support to initiatives lead by other organizations
Community engagement	49%	Working with community members and community organizations to build awareness around an issue, gain insight into an issue, and/or develop capacity within community members
Building partnerships	24%	Partnerships with individuals and organizations outside of the collaboration
External communication	23%	Efforts to communicate with public or other key stakeholders beyond members of the collaboration
Systems thinking	21%	Acknowledgement of complexity, multiple actors whose actions influence each other, delays between cause and effect, and/or interactions between system components
Engaging external experts	5%	Engaging experts external to the collaboration, such as scientists, policy experts, etc.
**Activities that influence or take place within the collaboration**		
Strategic thinking	28%	Discussion about where the collaboration is, where it is going, and how to get there
Sustainability efforts	23%	Plans, activities, or strategies discussed or implemented to increase the likelihood of sustained change in a community, or the sustainability of the collaboration
Capacity building	22%	Members have the opportunity to learn new skills and build capacity to address a complex problem
Quality improvement	6%	Description of monitoring and evaluating quality so that the collaboration or its activities can adapt as needed
**Evaluation continuum**		
Evaluate the collaboration	24%	Collect information about collaboration characteristics, such as structure, membership, and decision-making processes
Process evaluation	23%	Description of an evaluation focused on process, including implementation, satisfaction, attendance, and other measures; Process measures can be informed by theory or selected according to specific collaboration activities
Evaluate activities	34%	Collect information that helps collaboration members understand how an activity went, what it cost (financial or other), and/or what impact it had
Outcome evaluation	16%	Description of an evaluation that focused on the outcomes or impacts of collaboration activities, such as health, education, environmental, economic and other impacts
Intended outcomes	33%	Individual, community, system or other type of outcome that the collaboration is working towards or intending to influence

Note that codes were not mutually exclusive. Some excerpts were coded with multiple codes and therefore could have been used as examples for more than one code.

### Conceptual diagram

**Fig 2:**
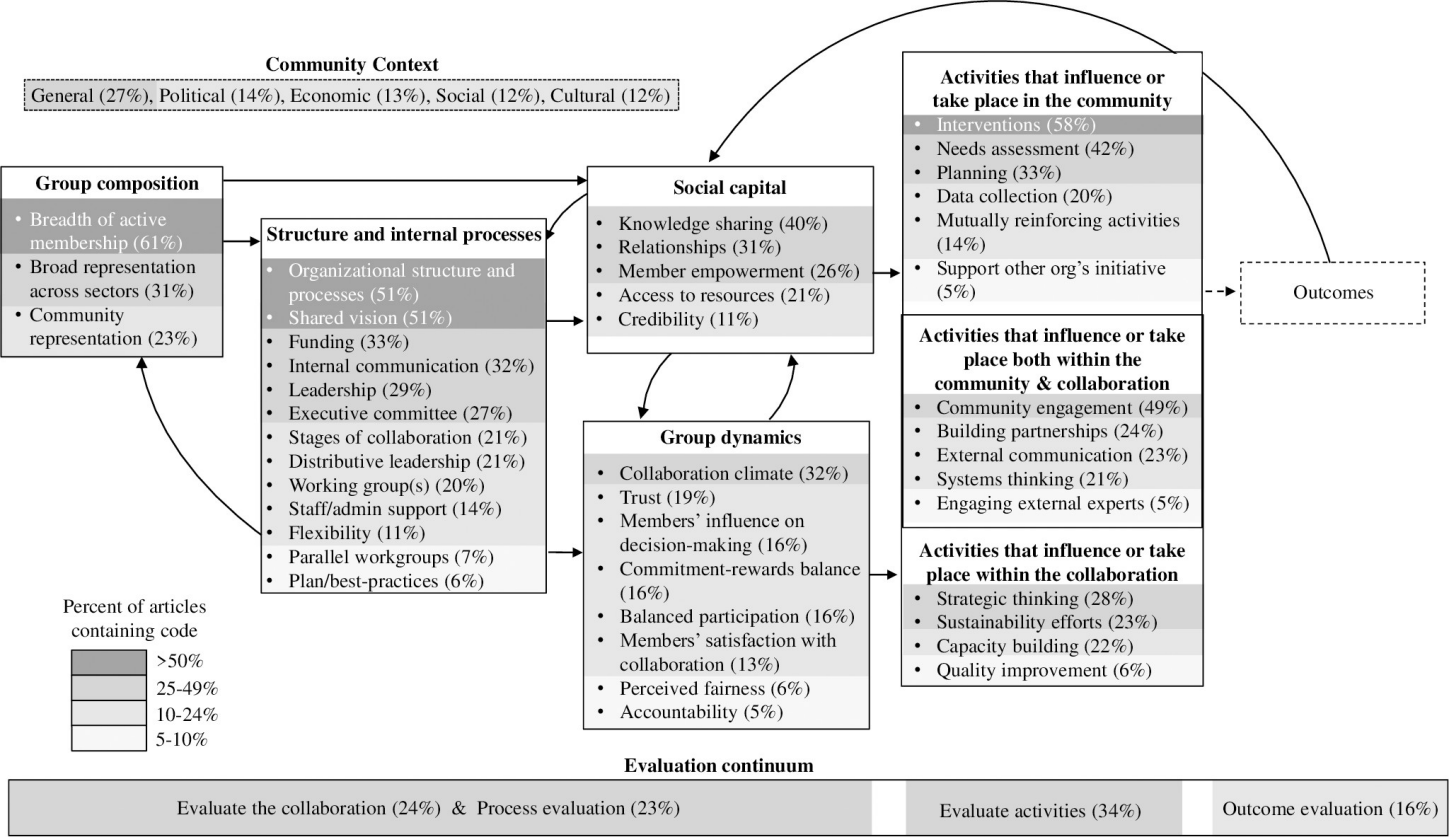
Consolidated Framework for Collaboration Research (CFCR) conceptual diagram synthesizing constructs that appeared in five or more of the articles included in the review.

We synthesized findings from this review in the Consolidated Framework for Collaboration Research (CFCR) (Fig 2). The domains in Table 2 directly map onto the domains and constructs presented in Fig 2. Domains include community context; group composition; structure and internal processes; group dynamics; social capital; activities that influence or take place within the collaboration; activities that influence or take place within community; and activities that influence or take place both in the collaboration and in the community. The CFCR is shaded to show code frequencies and organizes constructs into domains that theoretically influence one another as indicated with arrows, based on their timing or function within a collaboration. For example, structure and internal processes are ideally established early in a collaboration’s timeline and they help guide aspects of a collaboration’s group dynamics and social capital. Community engagement is integrated throughout the figure, including in the group composition and in activities that influence or take place within communities.

The CFCR acknowledges the role of context and evaluation opportunities within cross-sector collaboration work. Elements of community context influence all aspects of collaborations and are therefore depicted in a box with a dashed perimeter in the top left of the framework. An evaluation continuum spans the bottom of the figure. The continuum shows evaluation activities that align with the boxes above. Evaluation activities are internally focused on the left-hand side of the continuum and then move from proximal to community-level outcome evaluation activities, which are shown on the right-hand side of the continuum. CFCR includes feedback loops through which domains that occur later in a collaboration’s timeline, such as activities, can affect earlier collaboration conditions, such as group composition and social capital, which later affect activities. Community-level outcomes, such as changes in norms, perceptions, behaviors, environments, policies, systems, health outcomes, and community capacity are contained within a dashed box in Fig 2 because change in community-level or population outcomes are the ultimate goal of most cross-sector collaborations’ work; however their detailed coding was out of the scope of this review because these outcomes are inconsistently described in publications focused on collaboration model structure and would require further follow-up with authors.

## Discussion

We identified, described, and synthesized 95 articles’ theories, models, frameworks and principles for cross-sector collaboration into the Consolidated Framework for Collaboration Research (CFCR). This framework organizes constructs into seven domains: community context; group composition; structure and internal processes; group dynamics; social capital; activities that influence or take place within the collaboration; activities that influence or take place within the broader community; and activities that influence or take place both in the collaboration and in the community. The domains, particularly the distinction between activities that take place in collaboration and activities that influence the t he community, build upon existing cross-sector collaboration literature and add new concepts to help move the field forward. The constructs mentioned in the most articles were breadth of active membership, organizational structure and processes, shared vision, and interventions. These may be the most fundamental components of cross-sector collaborations. The CFCR can be used by researchers, practitioners, funders and collaboration members to conceptualize and name elements of collaboration and to consider how those elements, if strengthened, can improve collaboration. More broadly, the framework could be a useful tool when starting, maintaining, or evaluating a collaboration, since it provides a comprehensive view of collaboration elements. We also recommend considering how these constructs relate to each other and desired outcomes. More specifically, as a synthesis across multiple theories and frameworks, the CFCR offers an overarching typology from which researchers and practitioners can select and use the constructs to promote theory development about what works where and why across multiple contexts. Thus, it is a framework that provides flexibility for use across diverse settings, contexts, and topics.

Our study expands existing literature and reviews to provide a broad, unified framework of constructs that have been described and/or tested within the cross-sector collaboration literature and synthesizes these findings into a conceptual model. Our framework includes almost all the constructs present in the CCAT and Allen’s model, though CFCR includes more constructs, an updated organization of constructs, and is based on a systematic review identifying and integrating constructs from a broader body of research. Foster-Fishman and colleagues conducted a similar review of 80 articles in 2001 and proposed a framework detailing critical elements of collaborative capacity at four levels: member, relational, organizational, and programmatic capacity [21]. de Montigny and colleagues’ 2019 review examining cross-sector collaborations for social change to promote population health built upon the five conditions described in Collective Impact and added a new condition: collective learning [12]. Our review offers a more detailed inventory of constructs to consider for cross-sector collaboration design, maintenance, and evaluation and offers an example for how complex relationships between those constructs could be tested. In 2006, Zakocs and Edwards published a comprehensive review of the factors that are related to health coalition effectiveness [20]. Our review identified many of the same factors present in that study and added more constructs to the unified framework. Roussos and Fawcett (2000) reviewed the evidence for whether collaborative partnerships influence environmental changes, community-wide behavior changes, and population-level health indicators [2]. They found some evidence of collaborations’ impact within the 34 studies they reviewed but noted that evaluation of community and population-level outcomes is challenging, as is assessing causality between partnerships’ actions and community-level outcomes. Our review differed from those by Zakocs and Roussos in that we did not assess cross-sector collaboration effectiveness, but instead focused on synthesizing the concepts found within the existing cross-sector collaboration theories, models, frameworks and principles described in the published literature–a necessary step before future research can test models stemming from this more complete framework.

This study highlighted community engagement approaches employed by cross-sector collaborations, including involving community members as collaboration members and mobilizing community members around specific collaboration priorities. Involvement of community members as active partners in addressing health and social concerns have become increasingly valued because of the potential to increase relevance of research findings, increase community capacity to affect change long-term, and alleviate persistent health disparities in historically underserved communities [122–125]. A study of coalition health equity capacity found that coalitions can increase their capacity with on-going training and technical assistance [126]. Our findings suggest that community engagement is an essential aspect of many cross-sector collaborations, though the specific approaches and extent of engagement appear to vary widely. The variation is important for cross-sector collaborations to consider as they use the CFCR to guide their planning and evaluation efforts. For example, we found evidence of engagement strategies across a spectrum from consultation to shared leadership within cross-sector collaborations. The strategies across the spectrum all have a role in engagement, and collaboratives need to carefully consider and evaluate of each for their specific context.

Our study has limitations. We did not assess the relationship between theories, models, frameworks and principles and effectiveness at changing community-level outcomes because very few included articles tested such relationships [42, 79]. Our inclusion criteria captured articles that described models; articles that evaluated a collaboration’s effectiveness, but did not describe the coalition’s model, were excluded. For example, several Allies Against Asthma community coalition studies [127, 128] and a national evaluation of state coalitions aiming to reduce underage drinking [129] were excluded because the studies tested the collaborations’ impact on community-level outcomes but did not describe the collaborations’ models. Comparing and testing theories, models, frameworks and principles to determine which are most effective under specific circumstances is an area for future research. Recognizing the variation in and complexity of collaboration models, this research must be undertaken with methods capable of accommodating this complexity (e.g., mediation, moderation, and dynamics illustrated in Fig 2).

In this review, we identified constructs but did not analyze how constructs were combined or sequenced within articles, or how constructs related to specific collaboration objectives. Future research could test the relationships between constructs to elucidate the mechanisms through which collaborations influence change in their communities [130]. Systems thinking tools, such as causal loop diagrams (CLDs) and network analysis, are designed to accommodate complexity and could facilitate such analysis.

**Fig 3:**
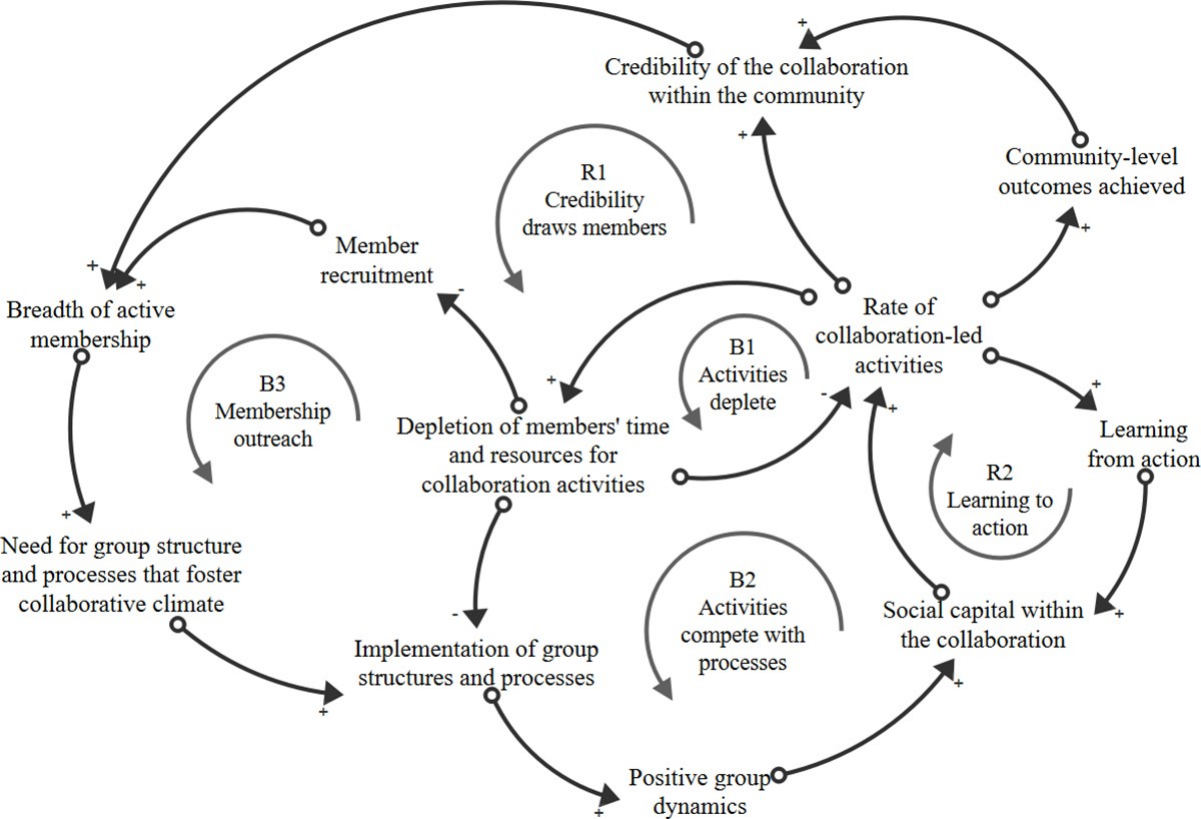
Causal loop diagram showing how several constructs identified in the review may relate to each other over time. In a CLD, a change in a variable at the tail end of an arrow is said to cause a change in the variable at the head end of that same variable, all else equal (e.g., an increase in the number of patrons at a popular restaurant leads to an increase in the wait time for a table, all else being equal). The direction of change is indicated by polarity symbol on the arrowhead. If a change in one variable (e.g., an increase) causes a change in the same direction for the other variable (e.g., it also increases), the polarity is positive (+), or said to be in the “same” direction (s). If a change in once variable causes a change in the opposite direction (e.g., an increase in one variable leads to a decrease in another variable), the polarity is negative (-) or said to be in the “opposite” direction (o). An important feature of CLDs is their ability to show feedback loops, or connections between variables where a chain of variables end up “feeding back” to the starting variable, and thus changing it. A critical CLD symbol is the nature of feedback loops, designated as either reinforcing (R) if the polarity within a feedback loops indicates that a change in one direction will be perpetuated throughout the loop, or as balancing (B) if changes within variables counteract each other, leading to a steady state or oscillation between states.

As an example of how systems thinking tools may be used, Fig 3 presents an illustrative CLD that shows hypothesized interactions between several constructs within CFCR described in reviewed articles. The CLD hypothesizes that breadth of active membership increases the need for group structure and processes, which can lead to positive group dynamics if the group processes are successfully implemented. Positive group dynamics can generate social capital within collaborations, leading to collaboration-led activities. However, as the rate of collaboration-led activities increases, members’ time and resources may become depleted and that can reduce the rate of collaboration-led activities (Fig 3, B1) and can reduce the implementation of group processes (B2). Depletion of collaboration members’ time may also reduce member recruitment initiatives, which can limit growth in the breadth of active membership (Fig 3, B3). The dynamics in this CLD begin to illustrate the complexity and interrelationships between constructs proposed by some of the articles included in this review, as well as in CCAT and Allen’s model (e.g., when coupling of constructs was recommended or one is described as setting the stage for or triggering another). Future research should test the relationships such as those in Fig 3 and other complex collaboration mechanisms to advance our understanding of not only *what* constructs are important for studying collaborations, but *how* those constructs are interrelated. Moreover, CLDs and a participatory approach to developing them called Group Model Building, can be used within collaborations to guide group members’ understanding of complex problems, and then to identify, prioritize, and learn about the potential impact of alternative actions designed to effect positive change [131–133].

## Conclusion

We conducted a systematic review of articles describing theories, models, frameworks and principles of cross-sector collaborations and synthesized our findings into the Consolidated Framework for Collaboration Research (CFCR). This review and the resulting CFCR extends prior work by showing constructs and community engagement strategies that are important to consider when creating, sustaining, funding or studying cross-sector collaborations. Fig 3 is an example of how dynamic relationships within collaborations can be diagramed and tested. Systems science tools, such as CLDs, can improve our understanding of how and why cross-sector collaborations may or may not function to influence health outcomes in their communities.

## Supporting information

S1 Checklist PRISMA 2009 checklist. (DOC)

S1 Table Systematic search conducted in PubMed. Equivalent searches were performed in Embase and EBSCO (CINHAL Plus with Full Text and Social Work Abstracts).
(DOCX)

S2 Table Construct codes and sample construct excerpts or excerpt summaries from articles included in the review. (DOCX)
