# Supplementary material for: Consolidated Framework for Collaboration Research derived from a systematic review of theories, models, frameworks and principles for cross-sector collaboration
Source: PLoS One. 2021 Jan 4;16(1):e0244501. doi: 10.1371/journal.pone.0244501 (PMC7781480; doi:10.1371/journal.pone.0244501)
Supplement: S1 Table — Equivalent searches were performed in Embase and EBSCO (CINHAL Plus with Full Text and Social Work Abstracts). (DOCX) [file pone.0244501.s002.docx]

S1 Table. Systematic search conducted in PubMed. Equivalent searches were performed in Embase and EBSCO (CINHAL Plus with Full Text and Social Work Abstracts).

| Logic | | Term location | Search terms |
| --- | --- | --- | --- |
| OR | And | Title | “Cross-sector network” OR “Cross-sector networks”  OR "Multisector network" OR "Multisector networks" OR "Multi-sector network" OR "Multi-sector networks"  OR "Multi-system collaboration" OR "Multi-system collaborations" OR "Multi-systems collaboration" OR "Multi-systems collaborations"  OR “Council" OR “Councils"  OR "Interagency coordination" OR “Inter-agency coordination” OR “Interagency collaboration” OR “Interagency collaborations” OR “Inter-agency collaboration” OR “Inter-agency collaborations”  OR "Coalition" OR "Coalitions"  OR "Cross sector collaboration" OR "Cross sector collaborations" OR "Cross-sector collaboration" OR "Cross-sector collaborations"  OR "Interorganizational relationships" OR "Inter-organizational relationships"  OR "Inter-organizational collaboration" OR "Interorganizational collaboration" OR "Inter-organizational collaborations" OR "Interorganizational collaborations"  OR "Cross-system collaboration" OR "Cross-system collaborations" OR "Cross system collaboration" OR "Cross system collaborations"  OR "Network Effectiveness" OR "Collective impact"  OR "Community collaborative" OR "Community collaboratives"  OR "Collaboration processes" OR "Collaboration process" |
|  |  | Title & Abstract | "Framework" OR "Frameworks" OR "Model" OR "Models" OR “Modeling” OR "Principles" OR "Theory" OR "Theories" |
|  | And | Title & Abstract | “Cross-sector network” OR “Cross-sector networks”  OR "Multisector network" OR "Multisector networks" OR "Multi-sector network" OR "Multi-sector networks"  OR "Multi-system collaboration" OR "Multi-system collaborations" OR "Multi-systems collaboration" OR "Multi-systems collaborations"  OR “Council" OR “Councils"  OR "Interagency coordination" OR “Inter-agency coordination” OR “Interagency collaboration” OR “Interagency collaborations” OR “Inter-agency collaboration” OR “Inter-agency collaborations”  OR "Coalition" OR "Coalitions"  OR "Cross sector collaboration" OR "Cross sector collaborations" OR "Cross-sector collaboration" OR "Cross-sector collaborations"  OR "Interorganizational relationships" OR "Inter-organizational relationships"  OR "Inter-organizational collaboration" OR "Interorganizational collaboration" OR "Inter-organizational collaborations" OR "Interorganizational collaborations"  OR "Cross-system collaboration" OR "Cross-system collaborations" OR "Cross system collaboration" OR "Cross system collaborations"  OR "Network Effectiveness" OR "Collective impact"  OR "Community collaborative" OR "Community collaboratives"  OR "Collaboration processes" OR "Collaboration process" |
|  |  | Title | "Framework" OR "Frameworks" OR "Model" OR "Models" OR “Modeling” OR "Principles" OR "Theory" OR "Theories" |
| Not | "animal" OR "animals" OR "animal model" OR "animal models" | | |
