# Supplementary material for: Consolidated Framework for Collaboration Research derived from a systematic review of theories, models, frameworks and principles for cross-sector collaboration
Source: PLoS One. 2021 Jan 4;16(1):e0244501. doi: 10.1371/journal.pone.0244501 (PMC7781480; doi:10.1371/journal.pone.0244501)
Supplement: S2 Table — (DOCX) [file pone.0244501.s003.docx]

S2 Table. Construct codes and sample construct excerpts or excerpt summaries from articles included in the review.

| **Construct code** | **Example excerpt** |
| --- | --- |
| **Community context** | |
| Community context | “Community Context - A pervasive factor for any coalition is the collective characteristics of the community where it strives to do its work. A set of community characteristics provides a backdrop for the coalition’s efforts and can either support or inhibit its success. It is, therefore, important for coalitions to pay attention to these characteristics, particularly during its formative stage” |
| Political context | “Macro- (government) system factors: Structure of government departments and agencies - The siloed structure of health, education and social service departments and agencies was found to impede service integration and the ability of providers to work collaboratively.” |
| Economic context | “ . . . the societal level consists of factors that create social and cultural norms, including the health, economic, educational, and social policies that help to maintain economic or social inequalities between groups in society” |
| Social context | “Facilitating the development of community infrastructures such as coalitions and providing training aimed at assisting communities in understanding the social and political context of problems and their potential solutions.” |
| Cultural context | “Coalition participants needed to be culturally competent and able to work comfortably with diversity. They needed to simultaneously respect boundaries and find ways to cross them.” |
| **Group composition** | |
| Breadth of active membership | “Breadth of active membership – the extent to which a range of stakeholder groups (e.g., law enforcement, judiciary, IPV advocates, healthcare) were actively represented on council steering and subcommittees” |
| Broad representation | “Almost by definition, community-based coalitions exhibit organizational and demographic diversity. Individuals representing different organizations often hold different perspectives on time, risk, and decision-making. They also often bring differing degrees of authority and differing preferences for communication and documentation. The demographic diversity that commonly results from efforts to achieve broad inclusion may also usher into the microcosm of the partnership conflict over larger, societal issues about race, class, ethnicity, wealth, and inequality” |
| Community representation | “The ICOH model considers that effective coalitions have two distinct features. First, they have a diverse community membership that works well together to achieve outcomes in each of the seven theoretical constructs…” |
| **Structure and internal processes** | |
| Organizational structure and processes | “A backbone support organization with staff who handle the administrative and logistical details associated with coordinating multiple organizations using adaptive leadership, facilitation, technology and communications support” |
| Shared vision | “Establish a clear vision and goals for the councils, both at a community and state level in order to insure their success and sustainability. Without these, and without revisiting them from time to time, council efforts will be uneven because they are dependent on the efforts of leadership that may vacillate due to demands and expectations of various organizations” |
| Funding | “While in-kind resources and volunteers significantly contribute to [coalition] efforts, funding is necessary to build capacity, momentum, and leadership at the local level” |
| Internal communication | “Formal and informal communication and cooperation between agencies affects awareness and the development of collaborative health initiatives” |
| Leadership | “Our revised model suggests that the influence of leadership and governance on participation works primarily through their positive relationship with vision consensus, which, in turn, influences perceptions of participation benefits and, subsequently, participation” |
| Executive committee | “The structure included a steering committee or coordinating council, and action committees for each community sector, such as schools, businesses, and health organizations” |
| Stages of collaboration | “Phases: 1) initiating action, 2) organizing for action, 3) sustaining action and impact” |
| Distributive/ empowerment leadership | “To achieve truly effective health partnerships, more widespread use of leadership models that distribute decision-making and authority across collaborators is necessary. This distributed-decision making maximizes contributions and engagement” |
| Working group(s) | “Single steering committee with various subcommittees organized by particular counties and/or by specific issues (e.g., law enforcement response, elder abuse; visitation centers; child witnessing)” |
| Staff/admin support | “Coalitions had a paid coordinator that filled a central role as the coalition convener, mobilizer and manager ensuring that coalition activities were aligned” |
| Flexibility | “Emphasis on flexibility has become paramount in today’s society marked by complexity and uncertainty” |
| Parallel working groups | “Working committees” |
| Adherence to a well-defined plan or best practice | “Develop a clear action plan and modify as needed” |
| **Social capital** | |
| Knowledge sharing | “Continuous communication among stakeholder groups to build trust and provide opportunities for the exchange of knowledge and expertise” |
| Relationships | “Our experience has shown that an overriding factor influencing the success of working in alliances is the nature of the relationships” |
| Member empowerment | “Member empowerment - degree to which members perceived being individually empowered to affect change (i.e., to influence policy and practice in the community) as a result of participation in the council” |
| Access to resources | “Collaborating helps gain catalyst, or start-up, funding, which can be used to attract more dollars to support a broader scope of activities” |
| Credibility | “Community support of the council depends upon the perception that the council is representing the community's needs which in turn affects the council's ability to recruit members” |
| **Group dynamics** | |
| Collaboration climate | “There is a sense of unity and cohesion in our board” |
| Trust | “Mutual trust” |
| Members’ influence on decision-making | “Decision-making processes that are collaborative enable extensive member involvement and help to ensure that particular interests do not dominate over the will of the collective during vision development” |
| Commitment-rewards balance | “Members expectations of payoffs/rewards - things members hope to obtain that they could not achieve taking action alone” |
| Balanced participation | “Vision consensus indicates shared purpose and alignment of interests in the coalition by enabling all members to achieve the desired benefits of participation without anyone disproportionately bearing the costs” |
| Members’ satisfaction with collaboration | “Satisfaction with planning process” |
| Perceived fairness | “Results showed that perceived personal influence in decision making, perceived clarity in decision-making processes, and perceived collaboration in conflict resolution demonstrated significant relationships with not only perceived procedural fairness but also with satisfaction with coalition decisions, personal engagement in the coalition, and organizational integration of coalition goals and activities.” |
| Accountability | “Creating Accountability - Members of a coalition are often oriented toward taking action and feeling accountable to other members. This motivational effect can be very important in facing and overcoming common implementation barriers such as administrative red tape hurdles and lack of leadership” |
| **Activities that influence or take place in the community** | |
| Interventions | “The council needs to be continuously engaged in meaningful projects and feel that their participation is impactful and creates change” |
| Needs assessments | “ The planning committee examined information on maternal and child health status, available health and social services, and social determinants (economic, educational and environmental) for four phases of the reproductive life-course: infancy, childhood and adolescence, preconception, and pregnancy and childbirth” |
| Planning | “Planning (create an action plan that links needs with specific strategies, the resources needed to implement them and the means of assessing progress)” |
| Data collection | “An assessment by the community of the community’s resources and built environment was essential. The central principle of the assessment process was that the community partners worked collaboratively at every stage of the assessment from deciding what resources would be assessed, to how the resulting information would be analyzed and disseminated. The approach allowed CHC and its partners to use these assessments as a vehicle for building community awareness, community capacity, and ultimately policy advocacy” |
| Mutually reinforcing activities | “Work together through established mechanisms to meet a common goal; align resources and efforts, have shared processes and rely on the support of each partner; ensure organizational mandates” |
| Support other organizations’ initiatives | “The council is actively involved with other organizations outside of the council” |
| **Activities that influence or take place in both the community and collaboration** | |
| Community engagement | “Coalitions discovered that they needed to build upon community experience and knowledge to be successful in their work. By meeting communities where they were, coalitions worked toward policy goals that more closely reflected residents’ needs, resources, and desires. By allowing the community to prioritize the direction of advocacy efforts, the coalitions achieved policy change through increased community involvement and support.” |
| Building partnerships | “Achieve sustainable funding for care through state/local government, business, community partnerships” |
| External communication | “Community-wide awareness campaigns elevated discussion of connections to health in housing, schools, and other key sectors for community health.” |
| Systems thinking | “Bring together individual programs & organizations, increase network and collaboration, integrate systems, produce more effective service delivery, improve patient outcomes” |
| Engaging external experts | “Coalitions found greater success in their efforts when they worked closely with experts in environmental science and environmental justice advocates, who provided sound scientific data, engaged in discussions about policy approaches and strategies, and assisted in crafting testimony to policymakers. Some residents became environmental experts in their communities, furthering the policy advocacy efforts of the initiative.” |
| **Activities that influence or take place within the collaboration** | |
| Strategic thinking | “Although prioritizing can be difficult, it is essential to be realistic and choose winnable battles with the highest positive impact on the population” |
| Sustainability efforts | “Institutionalization - Plan for institutionalization of new programs and practices (e.g., community involvement and dispersed leadership); Develop and implement a plan for financially sustaining the catalyst role of the coalition” |
| Capacity building | “Capacity building - capacity building refers to the readiness or ability of the coalition and its members to take action aimed at changing risk or protective behaviors and transforming community conditions and systems so that a supportive environment exists to sustain behavior changes over time” |
| Quality improvement | “Write and sign a new inter-agency agreement reflecting commitment to continuous quality improvement through this structure” |
| **Evaluation continuum** | |
| Evaluate the collaboration | “Evaluation of the [collaboration] is an ongoing process” |
| Process evaluation | “Identify key quality assurance data points within this best practice protocol that administrators would review together on a regular basis from a shared database to evaluate fidelity of implementation and to inform further multi-system refinements” |
| Evaluate activities | “Implementation and evaluation - 1) the implementation of REACH 2010 targeted activities and the assessment and acknowledgment of existing activities that are aimed at the community; 2) the implementation of targeted actions that are thought likely to bring about changes in the community and systems or changes among change agents; 3) change in widespread risk or protective behaviors in the community of focus; 4) reductions in health disparities; 5) other or unexpected outcomes; and 6) the examination and recognition by the coalition of external influences on the community” |
| Outcome evaluation | “As part of the goal of developing an evaluation proficiency at the local level of each community partnership, yearly evaluation plans are developed and implemented in each coalition community. Community-specific evaluation results are then compiled at the end of each year, and disseminated to local community members and leaders as appropriate. Evaluation results and reports have been used to seek local funding, write grant proposals and inform community leaders about the impact of partnership efforts” |
| Intended outcomes | “Community change . . . widespread behavior change; improvement in population level outcomes” |
